# Clinical characteristics of women with gestational diabetes - comparison of two cohorts enrolled 20 years apart in southern Brazil: Caraterísticas clínicas de mulheres com diabetes gestacional - uma comparação de duas coortes arroladas em intervalo de 20 anos no sul do Brasil

**DOI:** 10.1590/1516-3180.2016.0332190317

**Published:** 2017-08-07

**Authors:** Angela Jacob Reichelt, Letícia Schwerz Weinert, Livia Silveira Mastella, Vanessa Gnielka, Maria Amélia Campos, Vânia Naomi Hirakata, Maria Lúcia Rocha Oppermann, Sandra Pinho Silveiro, Maria Inês Schmidt

**Affiliations:** I MD, PhD. Physician, Division of Endocrinology, Hospital de Clínicas de Porto Alegre (HCPA), Porto Alegre (RS), Brazil.; II MD, PhD. Postgraduate Medical Sciences Program on Medicine, School of Medicine, Universidade Federal do Rio Grande do Sul (UFRGS), Porto Alegre (RS), Brazil.; III MD, MSc. Postgraduate Student, Postgraduate Medical Sciences Program on Medicine, School of Medicine, Universidade Federal do Rio Grande do Sul (UFRGS), Porto Alegre (RS), Brazil.; IV Medical Student, School of Medicine, Universidade Federal do Rio Grande do Sul (UFRGS), Porto Alegre (RS) Brazil.; V MD, MSc, Physician, Division of Endocrinology, Hospital Nossa Senhora da Conceição, Porto Alegre (RS), Brazil.; VI MSc. Statistician, Biostatistics Unit, Hospital de Clínicas de Porto Alegre (HCPA), Porto Alegre (RS), Brazil.; VII MD, PhD. Professor, Postgraduate Program on Gynecology and Obstetrics, School of Medicine, Universidade Federal do Rio Grande do Sul (UFRGS), Porto Alegre (RS), Brazil.; VIII MD, PhD. Professor, Postgraduate Medical Sciences Program on Medicine, School of Medicine, Universidade Federal do Rio Grande do Sul (UFRGS), Porto Alegre (RS), Brazil.; IX MD, PhD. Professor, Postgraduate Program on Epidemiology, School of Medicine, Universidade Federal do Rio Grande do Sul (UFRGS), Porto Alegre (RS), Brazil.

**Keywords:** Gestational diabetes, Pregnancy, Diagnosis, Pregnancy complications, Infant, newborn

## Abstract

**CONTEXT AND OBJECTIVE:**

The prevalence and characteristics of gestational diabetes mellitus (GDM) have changed over time, reflecting the nutritional transition and changes in diagnostic criteria. We aimed to evaluate characteristics of women with GDM over a 20-year interval.

**DESIGN AND SETTING:**

Comparison of two pregnancy cohorts enrolled in different periods, in university hospitals in Porto Alegre, Brazil: 1991 to 1993 (n = 216); and 2009 to 2013 (n = 375).

**METHODS:**

We applied two diagnostic criteria to the cohorts: International Association of Diabetes and Pregnancy Study Groups (IADPSG)/World Health Organization (WHO); and National Institute for Health and Care Excellence (NICE). We compared maternal-fetal characteristics and outcomes between the cohorts and within each cohort.

**RESULTS:**

The women in the 2010s cohort were older (31 ± 7 versus 30 ± 6 years), more frequently obese (29.4% versus 15.2%), with more hypertensive disorders (14.1% versus 5.6%) and at increased risk of cesarean section (adjusted relative risk 1.8; 95% confidence interval: 1.4 - 2.3), compared with those in the 1990s cohort. Neonatal outcomes such as birth weight category and hypoglycemia were similar. In the 1990s cohort, women only fulfilling IADPSG/WHO or only fulfilling NICE criteria had similar characteristics and outcomes; in the 2010s cohort, women only diagnosed through IADPSG/WHO were more frequently obese than those diagnosed only through NICE (33 ± 8 kg/m^2^ versus 28 ± 6 kg/m^2^; P < 0.001).

**CONCLUSION:**

The epidemic of obesity seems to have modified the profile of women with GDM. Despite similar neonatal outcomes, there were differences in the intensity of treatment over time. The IADPSG/WHO criteria seemed to identify a profile more associated with obesity.

## RESUMO

**CONTEXTO E OBJETIVO:**

Prevalência e características do diabetes *mellitus* gestacional (DMG) modificaram-se com o tempo, refletindo transição nutricional e diferentes critérios diagnósticos. Nosso objetivo foi avaliar características de gestações com DMG em intervalo de 20 anos.

**TIPO DE ESTUDO E LOCAL:**

Comparação de duas coortes gestacionais arroladas em diferentes períodos, em hospitais universitários de Porto Alegre, Brasil: 1991 a 1993 (n = 216) e 2009 a 2013 (n = 375).

**MÉTODOS:**

Aplicamos dois critérios diagnósticos às coortes: International Association of Diabetes and Pregnancy Study Groups (IADPSG)/Organização Mundial de Saúde (OMS); e National Institute for Health and Care Excellence (NICE). Comparamos características e desfechos materno-fetais entre as coortes e dentro de cada uma.

**RESULTADOS:**

Na coorte dos anos 2010, as mulheres eram mais velhas (31 ± 7 *versus* 30 ± 6 anos), obesas (29,4% *versus* 15,2%), apresentaram mais distúrbios hipertensivos (14,1% *versus* 5,6%) e risco aumentado de cesariana (risco relativo ajustado 1,8; intervalo de confiança de 95% 1,4 - 2,3), comparadas às da coorte de 1990. Desfechos neonatais, como categoria do peso ao nascer e hipoglicemia, foram semelhantes. Na coorte de 1990, essas características e desfechos foram semelhantes nas mulheres que preenchiam apenas um dos critérios; na de 2010, mulheres diagnosticadas apenas pelo IADPSG/OMS eram mais obesas (33 ± 8 kg/m^2^ *versus* 28 ± 6 kg/m^2^, P < 0,001) do que as diagnosticadas apenas pelo NICE.

**CONCLUSÃO:**

A epidemia de obesidade parece ter modificado o perfil de mulheres com DMG. Embora desfechos neonatais sejam semelhantes, houve diferenças na intensidade de tratamento ao longo do tempo. O critério da IADPSG/OMS parece identificar um perfil mais associado à obesidade.

## INTRODUCTION

Gestational diabetes (GDM), initially defined as the highest glycemic distribution values, has been surrounded by controversy, as detailed in the World Health Organization (WHO) position in 2013^1^ and illustrated in a timeline.^2^ From the 1980s to 2010, two general procedures were in vogue, one based on a 2 h/75 g oral glucose tolerance test (OGTT) with two plasma glucose values and diagnostic criteria similar to those used outside of pregnancy, and another one based on a 3 h/100 g OGTT, with four pregnancy-specific plasma glucose cutoffs.^1^

Screening for gestational diabetes in Brazil was infrequent before the 1990s, but both OGTT procedures were increasingly adopted thereafter. The 2 h/75 g OGTT gained wider acceptance after a 1997 consensus meeting^3^ at which GDM was defined using the intermediate hyperglycemic cutoffs that are used outside of pregnancy (fasting ≥ 110 mg/dl; 2 h ≥ 140 mg/dl). This definition was validated using data from the Brazilian Gestational Diabetes Study (Estudo Brasileiro de Diabetes Gestacional, EBDG)^4^ and remained the main diagnostic criterion used in Brazil, usually with two-step screening based on fasting values.^3^

In 2010, the International Association of Diabetes and Pregnancy Study Group (IADPSG) made new recommendations based on a 75 g OGTT and using data from the Hyperglycemia and Adverse Pregnancy Outcome (HAPO) study.^1^ Their recommendations have been endorsed by various entities, but new controversies arose. Perhaps the most important of these was the observed increase in GDM prevalence, especially when applied universally.^5^^,^^6^

This led other bodies to maintain the previous two-step diagnostic and screening procedures.^1^ In 2013, the World Health Organization (WHO) recommended the IADPSG criteria,^1^ although it warned of possible difficulties in implementing them. Alternatives to aid implementation were also proposed.^7^ In 2015, the British National Institute for Health and Care Excellence (NICE)^8^ made new recommendations. These are quite similar to the 1999 WHO^1^ criteria for the 2 h value (140 mg/dl), but specifically define a lower fasting plasma glucose cutoff (100 mg/dl) that matches the cutoff for impaired fasting glucose established by the American Diabetes Association in 2004.^9^ This strategy had been previously suggested by the Latin American Diabetes Association, in 2007,^10^ and resembles the one adopted in Brazil in 1997, although at that time, it was based on the impaired fasting glucose cutoff in vogue for use outside of pregnancy (110 mg/dl). Although the 1997 diagnostic criteria are still used in Brazil, the new IADPSG/WHO criteria are increasingly being adopted. The question that arises is whether the clinical profile of women detected through the IADPSG/WHO criteria differs from the profile of those detected through the NICE criteria. Moreover, it can be asked whether these profiles have changed over time.

## OBJECTIVE

The aim of the study was to evaluate changes in clinical characteristics and maternal and offspring outcomes over 20 years, between two Brazilian cohorts of women with GDM, and compare them when classified through the IADPSG/WHO or NICE criteria.

## METHODS

This study is a comparison of two cohorts of pregnant women in Brazil.

We studied two cohorts of women with GDM who had singleton pregnancies and at least one prenatal appointment in two university hospitals. A 75 g OGTT with two or three glucose measurements was available for 560 (94.8%) women, while confirmatory fasting plasma glucose data was available for 31 (5.2%). We applied two recent criteria for GDM to both cohorts:

1. the IADPSG/WHO criteria: FPG ≥ 92 mg/dl or 1-h plasma glucose ≥ 180 mg/dl or 2-h plasma glucose ≥ 153 mg/dl;^1^ and
2. the NICE criteria: fasting plasma glucose (FPG) ≥ 100 mg/dl or 2 h plasma glucose ≥ 140 mg/dl.^8^

The first cohort was composed of 216 women who met either of the two contemporary criteria for GDM (IADPSG/WHO or NICE). This cohort was derived from a cohort of 1031 women who were enrolled between 1991 and 1993, in general prenatal clinics of two university hospitals in Porto Alegre, which was one of the centers of the EBDG study.^4^ In the original cohort, cases with known pre-gestational diabetes had been excluded at the time of booking, and only the cases that reached diabetes levels outside of pregnancy had been treated.^11^

The second cohort was recruited between November 2009 and December 2013 and was composed of 375 women who had been referred to a high-risk pregnancy prenatal clinic at a public university hospital located in the southernmost state of the country, which provides medical care through the Brazilian National Health System (Sistema Único de Saúde, SUS). In 2013, around 3,800 babies were delivered at the hospital and the cesarean rate was 35.16%.^12^ All eligible women with singleton pregnancies who had a diagnosis of GDM through either of the two GDM criteria were included and cared for by a multidisciplinary team. The dietary counseling differed according to the individuals’ BMI and gestational stage, and emphasized low glycemic index and carbohydrates, along with high intake of fiber-rich foods.

We collected information on sociodemographic characteristics, medical history and pregnancy outcomes. The pre-gestational weight was obtained through self-reporting. Weights and heights were measured with the subjects wearing light clothes and no shoes. Use of diets, insulin and oral medications (metformin or glyburide) were considered to be “any treatment”. Data on pregnancy follow-up, delivery and maternal and newborn outcomes were retrieved from medical files.

A positive family history of diabetes was defined as among first-degree relatives, and gravidity, as the number of pregnancies including the current one. Pre-gestational BMI was calculated as the informed pre-pregnancy weight divided by the square of the height and categorized according to the current WHO classification.^13^ Total weight gain was calculated as the difference between the last registered weight (measured at delivery or at the last prenatal appointment) and the informed pre-pregnancy weight. The 2009 Institute of Medicine recommendations were used to classify weight gain adequacy: for underweight women, 12.5 to 18 kg; normal BMI, 11.5 to 16 kg; overweight, 7 to 11 kg; and obese, 5 to 9 kg.^14^ Hypertensive-related disorders of pregnancy were a composite of gestational hypertension, preeclampsia and eclampsia, as defined by the International Society for the Study of Hypertension in Pregnancy (ISSHP).^15^

We used the Alexander birth weight chart^16^ to classify newborns as small for gestational age (SGA) or as large for gestational diabetes (LGA), according to birth weight and gestational age. The latter was based on the first day of a reliable last menstrual period or on first-trimester ultrasonography. Macrosomia was defined as birth weight ≥ 4,000 g at term, and preterm birth, as delivery at less than 37 gestational weeks.^17^

The ethics committees of both hospitals approved the study protocols (number 90-058 for the 1990s cohort and number 10-0364 for the 2010s cohort). Informed consent was obtained from all individual participants included in the study.

### Statistical analysis

The data are presented as means (with standard deviation) or proportions (%). Student’s t test and Pearson’s χ^2^ test (with the Z test for comparison of proportions and Bonferroni’s correction) were used to compare the two GDM groups. Kappa statistics were used to calculate the level of agreement between the two diagnostic criteria. For adjustment of outcomes, we performed Poisson regression with robust variance and, in the models, we included the mothers’ baseline characteristics that were significant in univariable analyses. The outcomes assessed were: hypertensive disorders, cesarean section, preterm delivery, birth weight, frequencies of SGA and LGA, macrosomia, malformation, hypoglycemia and perinatal death. The 1990s cohort was taken to be the as reference and the results were presented as crude and adjusted relative risk (RR) and 95% confidence interval (CI). The statistical analyses were performed using the SPSS software, version 18.8. Statistical significance was set at 0.05, and was taken to be two-sided.

## RESULTS

The main characteristics of the two cohorts are shown in Table 1. Age, schooling and gravidity were greater in the recent cohort, while living with a partner and smoking decreased, the latter in a remarkable way (33.3 to 9.6%). The nutritional characteristics also changed importantly, such that the women of the 2010s cohort were notably more obese (45.1 versus 15.2%) before becoming pregnant and reached a higher weight at delivery (86 ± 18 versus 74 ± 12 kg). Accordingly, the plasma glucose values for the 2010s cohort were higher, based on fasting, 1 h and 2 h values. Additionally, the diagnosis of GDM was reached slightly earlier for the 2010s cohort and treatment was notably more frequent. The women of the 2010s cohort reported having markedly greater family histories of diabetes and having had a previous pregnancy with GDM. Although not statistically significant, a trend towards higher frequency of chronic hypertension was also observed, with slightly higher levels of diastolic blood pressure.

**Table 1:**
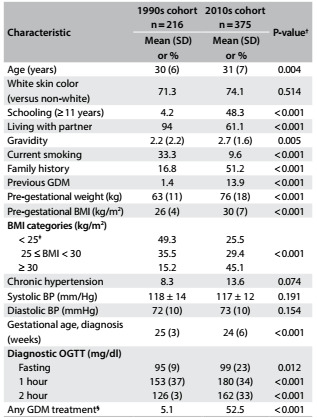
Characteristics of women in two gestational diabetes cohorts*, 20 years apart

BMI = body mass index; BP = blood pressure; GDM = gestational diabetes mellitus; OGTT = oral glucose tolerance test. *Gestational diabetes diagnosed through the criteria of either IADPSG/WHO (International Association of Diabetes and Pregnancy Study Groups/World Health Organization) or NICE (National Institute for Health and Care Excellence); ^†^Means (with standard deviation, SD) were compared using Student’s t test; proportions (%) were compared using Pearson’s χ^2^ test, with the Z test for proportions, adjusted using Bonferroni’s correction. ^‡^Includes one woman with BMI < 25 kg/m^2^ in each cohort; ^§^Any GDM treatment: diet for the 1990s cohort and diet + oral drug or insulin for the 2010s cohort.

As seen in Table 2, the women of the 2010s cohort ended their pregnancies with a slightly shorter duration and higher frequency of cesarean section. Pregnancy-related hypertension was more frequent but total gestational weight gain and adequacy of gestational weight gain did not differ much between the two cohorts. The main offspring outcomes were similar. Although not statistically significant, perinatal mortality decreased from 33/1,000 to 18/1,000. The adjusted relative risks of the main outcomes showed that there was higher risk of cesarean section in the 2010s cohort (Table 2). Although the difference in gestational age at delivery was significant in univariable analyses (5 days less in the 2010s cohort), the rates of preterm delivery were similar between the cohorts (14.9 versus 16.3%; P = 0.744).

**Table 2:**
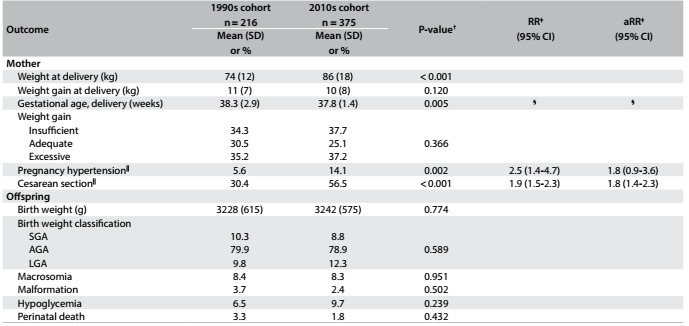
Maternal and offspring outcomes in two gestational diabetes cohorts*, 20 years apart

95% CI = 95% confidence interval; AGA = adequate for gestational age; aRR = adjusted relative risk; LGA = large for gestational age; RR = relative risk; SGA = small for gestational age. *Gestational diabetes diagnosed through the criteria of either IADPSG/WHO (International Association of Diabetes and Pregnancy Study Groups/World Health Organization) or NICE (National Institute for Health and Care Excellence). ^†^Means (with standard deviation, SD) were compared using Student’s t test; proportions (%) were compared using Pearson’s χ^2^ test, with the Z test for proportions, adjusted using Bonferroni’s correction. ^‡^Poisson regression with robust variance; ^§^See comment in Results section; ^||^Adjusted for center, age, schooling, gravidity, smoking, previous gestational diabetes mellitus (GDM) and pre-gestational body mass index (BMI).

**Figure 1:**
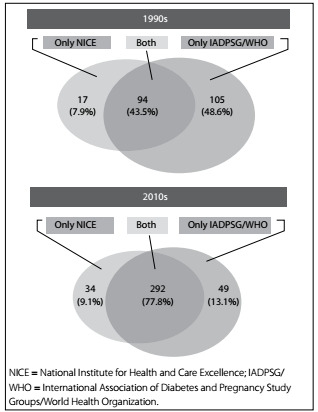
Overlap of NICE criteria and IADPSG/WHO criteria in two gestational diabetes cohorts 20 years apart

In the 1990s cohort, the NICE criteria would label 51.4% of women as having GDM, while the IADPSG/WHO criteria would label 94.5% as having GDM. In the 2010s cohort, 87.0% would meet the NICE criteria and 90.9%, the IADPSG/WHO criteria. The overall agreement between the two diagnostic criteria, examining the two cohorts together, was 68% (95% CI: 66-70%) but, as shown in Figure 1, the rate of agreement was greater for the 2010s cohort (43.5% in the 1990s cohort and 77.8% in the 2010s cohort). The proportion of the remaining cases that would be detected through only one of the two criteria decreased over time for those only meeting the IADPSG/WHO criteria (48.0% versus 13.1%) but not for those only meeting the NICE criteria.

We then compared the clinical characteristics and outcomes for women only meeting the NICE criteria or only meeting the IADPSG/WHO criteria for the two cohorts (Table 3). Although the numbers became small, it was apparent that women only meeting the IADPSG/WHO criteria had higher BMI and pre-gestational weight and showed a trend towards excessive gestational weight gain and delivery of heavier babies, but showed less neonatal hypoglycemia. Conversely, women only meeting the NICE criteria had higher rates of neonatal hypoglycemia. For both cohorts, the mean fasting plasma glucose was higher and the 2 h plasma glucose was lower for women who only met the IADPSG/WHO criteria.

**Table 3:**
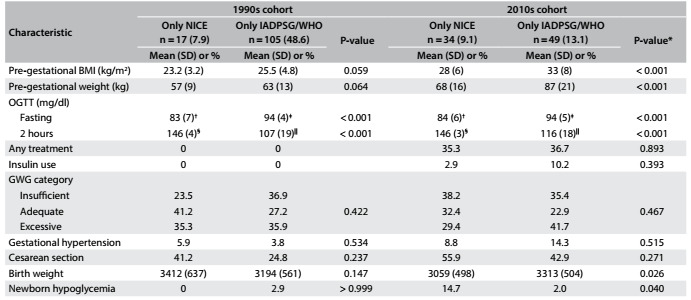
Characteristics and pregnancy outcomes of two gestational diabetes cohorts defined only through the NICE criteria or only through the IADPSG/WHO criteria

BMI = body mass index; GWG = gestational weight gain; IADPSG/WHO = International Association of Diabetes and Pregnancy Study Groups/World Health Organization; NICE = National Institute for Health and Care Excellence; OGTT = oral glucose tolerance test. Student’s t test for glycemic cutoffs: *Means (with standard deviation, SD) were compared using Student’s t test; proportions (%) were compared using Pearson’s χ^2^ test, with the Z test for proportions, adjusted using Bonferroni’s correction; ^†^P = 0.598; ^‡^P > 0.999; ^§^P > 0.999; ^|||^P = 0.006.

## DISCUSSION

The women in the more recent cohort of GDM were more obese, had higher plasma glucose values at diagnosis, higher frequency of pregnancy-related hypertension and higher adjusted risk of cesarean section than the previous cohort, which had been assembled about 20 years earlier. They were also more frequently treated for GDM. Newborn outcomes were similar over time, except for a downward trend in perinatal mortality. We found a very good overlap (Figure 1) between those diagnosed through the IADPSG/WHO and through the NICE criteria in the 2010s cohort. Women only meeting the IADPSG/WHO cutoffs showed a profile more associated with the effects of the ongoing obesity epidemic.

The differences observed between the two cohorts may reflect the nationwide public policies that have been adopted, which have resulted in better social indicators, as revealed by an increasing Human Development Index (from 0.608 in 1990 to 0.755 in 2014).^18^ This attainment is reflected in the higher schooling levels, later pregnancies (surprisingly, in contrast to higher gravidity) and better health indicators (such as lower rates of smoking)^19^ that have been seen in the whole Brazilian population.^18^ Furthermore, implementation of a national health system,^19^ which has enabled almost universal access to diagnosis and treatment for gestational diabetes, may have contributed, at least partly, to the differences found between the two cohorts.

However, the effects of the obesity epidemic have hampered these successes. Brazil moved up from 9^th^ position, in 1975, to 5^th^ position, in 2014, in the ranking of female obesity.^20^ Maternal obesity increased remarkably, revealed here through an average pre-gestational weight increase of 10 kg, in just 20 years between the two GDM cohorts. On average, the women in the 1990s cohort began pregnancy within the overweight category, whereas those in the 2010s cohort did so within the obesity category. Given that maternal obesity confers important adverse outcomes for both the mother and the child, and possibly for future generations,^21^ this epidemic rise in obesity threatens the progress in pregnancy outcomes that has already achieved. It also puts at risk the attainability of the goals for reducing the burden of non-communicable diseases by 2025, a challenge faced by Brazil and all other nations.

Hyperglycemia and obesity share common metabolic pathways and characteristics, and thus lead to consequences that are probably indissoluble, with additive effects on GDM outcomes.^22^ It is apparent that the effects of the obesity epidemic were fully manifested in our current cohort: the women were remarkably more obese, presented pregnancy hypertension more often and were at higher risk of cesarean section. Birth weight and the large-for-gestational-age rate among the newborns did not differ between the two cohorts, perhaps because of the more widespread treatment for GDM in the recent cohort.

Within this scenario, over the last few years, we have faced the challenge of adopting new diagnostic criteria, following the new recommendations from IADPSG in 2010 and WHO in 2013. Our main concern is that these criteria are likely to increase the prevalence of gestational diabetes,^23^ both as a result of the epidemic of maternal obesity and as a consequence of only requiring one altered cutoff for a diagnosis of GDM. Previous estimates indicated that changing from the 1997 Brazilian criteria to the new IADPSG/WHO criteria would raise the frequency of GDM from 7.6% to 18.0%, i.e. a 2.5-fold increase.^24^

As illustrated in Figure 1, by applying each criterion to the diagnostic test for women with GDM, the IADPSG/WHO criteria labeled a higher number of women in both cohorts as presenting GDM, although the rate of disagreement between the two criteria was lower in the 2010s cohort (down from 56.5% to 22.2%). The rate of agreement between the two different criteria varies across studies, from 49.7%^25^ or 50.6%^26^ to 65.6%.^27^ This partial overlap suggests that these studies probably reflect distinct GDM profiles. In one study that compared the NICE and the IADPSG/WHO criteria, 55.1% of the women with GDM would be detected by both criteria, which was lower than the overlap that we found in the 2010s cohort.^28^ It is possible that differences we found for the 2010s cohort concerning maternal weight and birth weight reflected the effects of the current obesity epidemic and associated factors, particularly in relation to those only meeting the IADPSG/WHO criteria. In a recent study comparing obese women with and without GDM in the first trimester of pregnancy, obesity markers such as insulin resistance and higher BMI were more frequent in those with GDM, along with higher glucose levels. It was suggested that application of IADPSG/WHO to the DALI cohort had “identified a profile akin to the metabolic syndrome”.^29^ Moreover, to be worthwhile, adoption of a GDM criterion that enhances prevalence should also increase the detection rate of relevant clinical outcomes. Given the low attributable fractions relating to hyperglycemia (6.7% for large for gestational age and 3.5% for preeclampsia, based on the IADPSG/WHO criteria),^24^ increased detection of relevant outcomes is likely to be small.

The main strength of our study is that it enables comparison between the features of a recent GDM cohort with those of an old one. We were able to document the important effect of the obesity epidemic over this 20-year interval. The major limitation of our study relates to the source of the cohorts: the 1990s cohort was derived from a large sample and had little intervention for treatment, and although the study was directed from university hospitals, the women were attending general prenatal care. On the other hand, for the 2010s cohort, enrollment was at a specialized clinic of a university hospital and women with greater severity of hyperglycemia may have been included. These women more frequently presented histories of family diabetes and previous GDM. This could have biased our results; nevertheless, diabetes rates are also increasing worldwide^30^ and this trend could potentially explain these findings. Intensive treatment in the 2010s cohort limited interpretation of pregnancy outcomes. Finally, only a few of our cases met only one criterion or the other, which limited the extrapolation of our data. Even so, some subtle differences were revealed.

## CONCLUSION

Important effects reflecting the nutritional transition over time were documented through evaluation of these two GDM cohorts separated by a 20-year interval, and some differences in applying two different GDM criteria were apparent. Women only meeting the IADPSG/WHO criteria presented pregnancy features that were often linked to obesity, while those meeting the NICE criteria presented worse neonatal outcomes, here represented by hypoglycemia. Further studies focusing on the combined effects of the obesity epidemic and hyperglycemia will help to clarify similarities and differences, and whether these are real, in the profile of pregnancies diagnosed through these two currently used GDM criteria.
